# BSHI/BTS guidance on crossmatching before deceased donor kidney transplantation

**DOI:** 10.1111/iji.12558

**Published:** 2021-09-23

**Authors:** S. Peacock, D. Briggs, M. Barnardo, R. Battle, P. Brookes, C. Callaghan, B. Clark, C. Collins, S. Day, N. Diaz Burlinson, P. Dunn, R. Fernando, S. Fuggle, A. Harmer, D. Kallon, D. Keegan, T. Key, E. Lawson, S. Lloyd, J. Martin, J. McCaughan, D. Middleton, F. Partheniou, A. Poles, T. Rees, D. Sage, E. Santos‐Nunez, O. Shaw, M. Willicombe, J. Worthington

**Affiliations:** ^1^ Tissue Typing Laboratory Cambridge University Hospitals NHS Foundation Trust Cambridge UK; ^2^ H&I Laboratory NHSBT Birmingham Vincent Drive Birmingham UK; ^3^ Clinical Transplant Immunology Churchill Hospital Oxford UK; ^4^ H&I Laboratory SNBTS Edinburgh UK; ^5^ H&I Laboratory Harefield Hospital Harefield UK; ^6^ Department of Nephrology and Transplantation Guy's Hospital London UK; ^7^ H&I Laboratory Leeds Teaching Hospitals NHS Trust UK; ^8^ H&I Laboratory Southmead Hospital Bristol UK; ^9^ Transplantation Laboratory Manchester Royal Infirmary Manchester UK; ^10^ Transplant Laboratory Leicester General Hospital Leicester UK; ^11^ H&I Laboratory, The Anthony Nolan Laboratories Royal Free Hospital UK; ^12^ Organ Donation & Transplantation NHSBT Stoke Gifford Bristol UK; ^13^ H&I Laboratory NHSBT Barnsley Centre Barnsley UK; ^14^ H & I Laboratory Royal London Hospital London UK; ^15^ Department of H&I Beaumont Hospital Dublin UK; ^16^ Organ Donation and Transplantation NHSBT Birmingham UK; ^17^ Welsh Transplantation & Immunogenetics Laboratory Cardiff UK; ^18^ H&I Laboratory Belfast Health and Social Care Trust Belfast UK; ^19^ H&I Laboratory Liverpool Foundation Trust Liverpool UK; ^20^ H&I Laboratory University Hospitals Plymouth Plymouth UK; ^21^ H&I Laboratory NHSBT Filton Bristol UK; ^22^ H&I Laboratory NHSBT Tooting Centre London UK; ^23^ H&I Laboratory Imperial College Healthcare NHS Trust London UK; ^24^ H&I Laboratory Viapath, Guys & St Thomas London UK; ^25^ Department of Immunology and Inflammation Imperial College London UK

**Keywords:** antibodies, HLA, kidney transplantation, virtual crossmatching

## Abstract

All UK H&I laboratories and transplant units operate under a single national kidney offering policy, but there have been variations in approach regarding when to undertake the pre‐transplant crossmatch test. In order to minimize cold ischaemia times for deceased donor kidney transplantation we sought to find ways to be able to report a crossmatch result as early as possible in the donation process. A panel of experts in transplant surgery, nephrology, specialist nursing in organ donation and H&I (all relevant UK laboratories represented) assessed evidence and opinion concerning five factors that relate to the effectiveness of the crossmatch process, as follows: when the result should be ready for reporting; what level of donor HLA typing is needed; crossmatch sample type and availability; fairness and equity; risks and patient safety. Guidelines aimed at improving practice based on these issues are presented, and we expect that following these will allow H&I laboratories to contribute to reducing CIT in deceased donor kidney transplantation.

## 1 INTRODUCTION

This document has been prepared by a working group set up at the request of the British Transplantation Society (BTS). The workshop, organized by the British Society for Histocompatibility and Immunogenetics (BSHI), was convened on 22 March 2019 in order to provide guidance on crossmatching for kidney transplantation from deceased donors in the United Kingdom. The brief was to resolve differences in crossmatch practices in relation to when a result becomes available (but not how crossmatching is performed). The overall aim was to minimize cold ischaemia time (CIT) and improve patient outcomes by agreeing ways to enable a crossmatch test to be performed as early as possible in the donation process.

The United Kingdom has a single kidney offering policy that all transplant units follow (ODT Policy POL186/10, 2019): we encourage the reader to be fully appraised of this document and consider it when interpreting the following guidance. In this scheme, deceased donor kidneys are offered according to rule‐based procedures, being regarded as a national asset while dependent on consent. The policy has been driven by notions of equality, equity and utility, and in later years organ offering has attempted to find the acceptable balance between the latter two (Courtney & Maxwell, 2009). Donor organ offering policies change with time due to developments in evidence, politics, donation rates and waiting list demographics. There has been a drift away from utility (e.g. HLA matching) and more towards equity (e.g. to reduce variations in waiting time). How the UK kidney offering schemes have evolved has been reviewed recently (Watson et al., 2020).

The current UK offering scheme was introduced in September 2019 and involves complex, evidenced‐based algorithms to give priorities to waiting time and level of antibody sensitization to HLA (calculated reaction frequency, cRF or panel reactive antibodies, PRA). These two factors are of course related; highly sensitized patients have tended to wait longer than less sensitized patients. This means less concern with HLA mismatches, which is supported by evidence showing a reduced influence of HLA matching on transplant outcomes (Su et al., 2004). The waiting list patients are in one of two tiers. Patients with a cRF of 100% (99.5% or greater), or those with a waiting time of seven or more years, or with a matchability (measure of difficulty to match relative to the previous 10,000 deceased donors) score of 10 are included in Tier A, the top priority. The remainder are included in Tier B and offering is then determined by an accumulated point score taking into consideration: donor–recipient risk index combinations; waiting time from earliest of dialysis or activation on the list; tissue match and age combined; location; matchability; total mismatch and blood group match. This applies to kidneys from both circulatory death (DCD) and brain death (DBD) donors.

Increased organ offering to highly sensitized patients and a greater proportion of DCD donors raises the need to improve our systems for delivering accurate, effective and timely pre‐transplant crossmatching. Particularly with the increased proportion of DCD donors, where cold ischaemia time less than 12 h is a recognized determinant of outcome (Summers et al., 2010), there is a benefit in reducing the time from offering to transplantation, and how we undertake the crossmatch can contribute to the extent of this period (Taylor et al., 2010). Irrespective of the details of the allocation or offering policy, the process of crossmatching begins once a specific, potential recipient has been identified. This process involves getting the right people, the right materials and the right information together at the right time, and these guidelines are concerned with optimizing that process.

Safe renal transplantation requires, amongst other things, pre‐transplant assessment of antibody compatibility (meaning an absence of antibodies that are harmful, either directly or by virtue of their being a biomarker of poor outcome). Compatibility can be determined directly by testing the reaction in a crossmatch (XM) between recipient serum and donor cells, typically leucocytes (LXM). The LXM can be performed using cells from donor lymph nodes or spleen, or donor peripheral blood lymphocytes (PBLs). Lymph nodes and spleen are only available after organ retrieval, whilst PBLs can be taken from the potential organ donor prior to removal of the kidneys. Alternatively, HLA antibody compatibility can be assessed by prediction when a recipient's HLA‐specific antibodies have been sufficiently characterized and the donor's corresponding HLA alleles have been identified (the virtual crossmatch, VXM).

All UK H&I laboratories regularly test waiting list patient samples from the transplant units they support (routine three monthly samples, BSHI & BTS Guideline, 2015) and the results are reported to the central deceased donor organ offering agency (NHSBT, ODT). Initial kidney offering also includes a computer algorithm that compares the listed unacceptable antigens (mostly based on antibody specificities) with the donor HLA type: essentially a virtual crossmatch. This minimizes the chance of a subsequent positive crossmatch following the donor offer and allocation.

Because the decision to proceed with deceased donor kidney transplantation cannot be made before the HLA XM result is reported, many of the planning decisions leading to and allowing the transplant to take place also cannot be made until this result is available. Timely XM reporting is therefore an essential component of an effective transplantation system where there is national sharing of deceased donor kidneys.

The introduction of the lymphocytotoxic crossmatch assay (Patel & Terasaki, 1969) was a step change in the field of transplantation as this demonstrated an increased incidence of hyperacute rejection in recipients with a positive complement dependent lymphocytotoxic crossmatch. Over the decades, improvements in laboratory techniques, immunosuppression and clinical management of both recipients and donors have led to year‐on‐year improvements in both graft and patient survival (Collaborative Transplant Study (CTS) K‐14101‐0820; Collaborative Transplant Study (CTS) K‐14102‐0820). Challenges still remained on the length of CIT for organs and the contribution of CIT to delayed graft function (DGF) in transplant recipients (Perico et al., 2004; Salahudeen et al., 2004; Siddiqi et al., 2004). Implementation and development of the H&I laboratory's pre‐transplant LXM testing have, along with other factors such as timely access to theatres, helped make CIT a modifiable factor which directly reduces DGF (Kolonko et al., 2011; Shrestha et al., 2016; Vacher‐Coponat et al., 2007).

In order to address this, laboratories began to consider whether there were certain scenarios in which they could predict, with sufficient confidence, the absence of damaging IgG HLA‐specific antibodies directed against donor mismatches (Taylor et al., 2000). Early adopters of this were the Cambridge Transplant Unit (Taylor et al., 2010) who referred to a pre‐transplant compatibility assessment without the need for day of transplant testing as a ‘virtual crossmatch’. In these early publications, the recipient groups were carefully defined with the majority being males with no sensitizing events and negative for HLA‐specific antibodies. Other centres adopted VXM policies and there are many publications in the literature across the world detailing different approaches to this in order to minimize CIT (Jani et al., 2017; Rohan et al., 2020; Turner et al., 2019). The work from Taylor et al. (2000) suggests a saving in CIT of at least 3 h is achievable if a VXM is used instead of a crossmatch requiring donor leucocytes, and this was also associated with a reduced incidence of delayed graft function. A heightened interest in VXM developed in the United States in 2014 with the implementation of the new Kidney Allocation Scheme which increased sharing of organs across greater distances (Morris et al., 2019). Similarly, in the United Kingdom, the kidney offering scheme updated in 2019 gives national priority to highly sensitized patients (HSPs) with shipping of kidneys from DCD and DBD donors across the country (ODT Policy POL186/10, 2019). Availability of a VXM has also helped adoption of the kidney fast‐track offering scheme in the United Kingdom, although this has tended to favour the less sensitized patients (Callaghan et al., 2017).

The detection of IgG HLA‐specific antibodies has evolved through flow cytometry, ELISA, Luminex screening and single antigen bead (SAB) assays, as summarized in a review by South and Grimm (2016). The increasing sensitivity of antibody detection has concurrently required a more detailed understanding of the limitations of solid phase assays, namely the presence of cryptic epitopes; non‐specific binding; high‐dose hook effect and differences between manufacturers (Kumar et al., 2021; Middleton et al., 2014; South & Grimm, 2016) because these are used to predict compatibility. Antibody assays based on detection against single antigens are clearly a significant enabler of general use of the virtual crossmatch. In part this is because of their sensitivity (the confidence in the assignment of negative, or null reactions) and also because this overcomes the inaccuracies in interpretation due to the confounding effect of multiple antigens and linkage disequilibrium seen with cell panel detection (Bingaman et al., 2008). This certainly requires an in‐depth understanding of the shortcomings of the sources of error, but overall, we should be assured that we have the tools to make the VXM a safe practice, in part because there is likely to be a bias towards caution. Hence the unresolved matter of the definition of a positive threshold measurement, a critical issue because the difference between negative and positive will influence both clinical outcome and access to transplantation. There is a range of threshold values used in different HLA laboratories for bead assays and with no consensus, but attempts are being made define more objective measures of positivity as well as understand the relationships between measured levels and outcomes (Wisse et al., 2019; Zecher et al., 2018). These issues also apply to other ways of detecting and measuring donor‐specific antibodies that have not been formally, clinically validated.

Parallel to this, HLA typing methodologies have evolved with the introduction of rapid DNA‐based testing, such as PCR‐SSP, PCR‐SSOP and subsequently, real‐time PCR (or q‐PCR), which permit typing of HLA‐A, ‐B, ‐C, ‐DRB1/3/4/5, ‐DQ and –DP, enabling a detailed characterization of HLA for deceased donors in a rapid timeframe. More recently, methodologies that can provide DNA sequencing in the required time frame have been developed (De Santis et al., 2020), including full length HLA gene sequencing (Stockton et al., 2020). The latter approach has the potential to eliminate all interpretation ambiguities. Within the United Kingdom, the field has reached a stage where donors are required to be characterized at least for HLA‐A, ‐B, ‐C, DRB1/3/4/5, ‐DQB1 and ‐DPB1 (ODT datasheet DAT2885/3, 2019).

While the majority of UK laboratories now offer the VXM, there may be differences between laboratories in both the acceptance criteria and practice for patient management to support using a VXM. Some will undertake a truly virtual (in silico) XM with no day of transplant sample testing required. Others will perform a day of transplant Luminex antibody test to facilitate a VXM. In all situations, the effective use of a VXM is dependent on correct and adequate donor HLA typing together with up to date information regarding the potential recipient's HLA antibody screening and sensitization history (Sullivan et al., 2018; Kumar et al., 2021). There were initial concerns that the exclusive use of sensitive assays such as SAB to facilitate a VXM may lead to excessive waiting times in the highly sensitized patient population (Meier‐Kriesche & Kaplan, 2002) as lower immunological risk recipients may be transplanted preferentially. More recent publications demonstrate a VXM is possible in these patient groups (Jaramillo et al., 2020; Turner et al., 2019; Valentin et al., 2016) or may actually increase access to transplantation for highly sensitized patients (Bingaman et al., 2008).

Scenarios where a VXM may not be possible include situations where a patient lacks up to date screening (Roll et al., 2020); where there is insufficient information about sensitizing events to be able to accurately predict risk; those highly sensitized patients with particularly complex HLA antibody profiles (Turner et al., 2019) and the situations where all the relevant HLA loci have not been sufficiently characterized in the donor. Essentially, this is dictated by the confidence in being able to predict antibody compatibility (Taylor et al., 2000). The BSHI & BTS guideline document (BSHI & BTS Guideline, 2015) describes circumstances where use of a VXM may or may not be appropriate. For those cases where there is insufficient confidence to use a VXM it is still important to minimize CIT due to the detrimental impact this has on the organ (Shrestha et al., 2016), and this could be achieved by undertaking a LXM using pre‐donation PBLs. Irrespective of the manner of crossmatching, the H&I laboratory should always request confirmation from the transplant unit that no potential sensitizing event has occurred since or just before the date of the last available sample. When a prospective LXM is used and found to be positive (due to HLA antibodies), it is essential that this has been determined as soon as possible (ideally prior to organ retrieval) to facilitate timely re‐offering of the kidney to other patients on the national transplant waiting list for deceased donor kidneys.

The bead and leucocyte binding assays are profoundly different, not just in terms of sensitivity. Both assays have been designed and validated for the detection of HLA‐specific antibodies but may be subject to non‐HLA reactivity. In particular, leucocytes carry an additional range of allo‐ and auto‐antigens, many unknown, and generally with their clinical significance untested. For this reason, a retrospective LXM of a negative prospective VXM test may, on occasions, be positive, and this will be due to non‐HLA. Such a retrospective test will of course not influence donor allocation, and we would welcome a formal, multi‐centre assessment of the value of retroactive verification of the VXM.

A pre‐transplant crossmatch to detect non‐HLA (and non‐ABO)‐specific antibodies is currently problematic for two main reasons. If a target cell is used, then this must be known to express the relevant antigen or antigens. This approach has been tested for potential endothelial cell markers but the evidence that such a test is predictive of rejection or transplant outcome is mixed (Breimer et al., 2009; Zitzner et al., 2013; Daniel et al., 2016). In addition, the cells used in the crossmatch, whether third party or a precursor in the endothelial cell lineage, may not fully represent the antigen load of the donor's actual graft endothelium. Antibodies specific to the human neutrophil antigen (HNA) system have also been implicated in transplant rejection. Both T and B lymphocytes are known to express HNA‐3, and antibodies against this can cause a positive crossmatch in the absence of HLA antibodies (Key et al., 2019). HNA‐3 sensitization is pregnancy associated, so these antibodies are mostly restricted to parous individuals. Others have shown that a positive LXM can be associated with the presence of certain non‐HLA autoantibodies, but it is not evident that these are the cause of the crossmatch reaction, and rejection is only seen in a minority of such cases (Kang et al., 2021).

The second problem lies with being able to define a positive XM result in the absence of a known, corresponding antigen. For autoantibodies, incompatibility can be assumed. For non‐HLA (and non‐ABO) alloantibodies, a specific incompatibility cannot be deduced as, currently, deceased donors are only typed for HLA, ABO and Rh. Pre‐transplant testing of these antibodies, whether allo or auto, is also required to reliably interpret a XM, and essential for a VXM, but this is not routinely undertaken in the United Kingdom as the level of evidence was insufficient at the time of development of the relevant guidelines (BSHI & BTS Guideline, 2015; Tait et al., 2013). Transplanting across unknown, non‐HLA antibodies is therefore the general, current practice in the United Kingdom. There are anecdotal reports of hyperacute rejection being associated with non‐HLA and non‐ABO incompatibilities, such as those involving other red cell‐specific antibodies (Shaw et al., 2019), but this is rare. Treatment of rejection associated with non‐HLA antibodies can be effected by the same agents and procedures used against HLA antibody‐mediated rejection (reviewed by Kardol‐Hoefnagel & Otten, 2021).

This guideline has been created to help each laboratory optimize their own processes in order to reduce CIT and maximize the chance of an allocated donor organ proceeding to transplantation. This is based on evidence and opinion concerning safety, efficient use of resources and logistics, effective use of all donor organs and fairness and equity of access to transplantation. Although written specifically for deceased donor kidney transplantation, the principles included are also applicable to other forms of transplantation where pre‐transplant assessment of antibody compatibility is required, for example simultaneous kidney and pancreas transplantation. Furthermore, the objectives underlying kidney allocation will be subject to future development. Indeed, the most recent UK offering scheme was published soon after these guidelines were prompted. However, offering changes are unlikely to determine the validity of this document as the guidance relates to events following donor offering, and a review of the current scheme in this context confirms this. The technical details of how crossmatches are actually performed were not reviewed by the working group and so are outside the scope of this guideline.

## 2 GRADING OF RECOMMENDATIONS

These guidelines represent consensus opinion from experts in the field of transplantation in the United Kingdom and represent a snapshot of evidence available at the time of writing. It is recognized that some recommendations are made even when the evidence is weak. It is felt that this is helpful to clinicians in daily practice.

In these guidelines the Grading of Recommendations Assessment, Development and Evaluation (GRADE) system has been used to rate the strength of evidence and the strength of recommendations (Atkins et al., 2004). The approach used in producing the present guidelines is consistent with that adopted by Kidney Disease Improving Global Outcomes (KDIGO) (Uhlig et al., 2006). Explicit recommendations are made on the basis of the trade‐offs between the benefits on one hand, and the risks, burden and costs on the other.

For each recommendation the quality of evidence has been graded as A (high), B (moderate), C (low) or D (very low):

**Grade A** evidence means high‐quality evidence that comes from consistent results from well‐performed randomized controlled trials or overwhelming evidence of another sort (such as well‐executed observational studies with very strong effects).
**Grade B** evidence means moderate‐quality evidence from randomized trials that suffer from serious flaws in conduct, consistency, indirectness, imprecise estimates, reporting bias or some combination of these limitations, or from other study designs with special strength.
**Grade C** evidence means low‐quality evidence from observational evidence, or from controlled trials with several very serious limitations.
**Grade D** evidence is based only on case studies or expert opinion.

For each recommendation, the strength of recommendation has been indicated as one of:

Level 1 (we recommend)
Level 2 (we suggest)

Not graded (where there is not enough evidence to allow formal grading)

A **Level 1** recommendation is a strong recommendation to do (or not to do) something where the benefits clearly outweigh the risks (or vice versa) for most, if not all patients.

A **Level 2** recommendation is a weaker recommendation, where the risks and benefits are more closely balanced or are more uncertain.

## 3 OBJECTIVES AND RECOMMENDATIONS

Objective 1. Address the impact of the reporting time of a crossmatch on the overall time in the transplant pathway. The key issues identified are, first, where a prospective crossmatch is required; a transplant cannot proceed until said crossmatch is completed. Second, in the United Kingdom, the positive crossmatch rate is around 2.5% (based on data between 2010 and 2015) (ODT Statistics and reports) and the organs in these cases require re‐offering and potentially a further crossmatch. Third, there are logistical issues around access to theatres for transplantation with kidney transplantation not always being given sufficient priority for operating theatre availability. Therefore, the availability of a crossmatch result ahead of organ arrival is an aid to surgical planning.

### 3.1 Recommendations

1. To overcome this, we recommend that a crossmatch result (either VXM or LXM) for a particular recipient must be reported before the organ arrives at the transplant centre, both LXM and VXM procedures will allow this (Grade 1B).
2. If a VXM is not possible then we recommend that where possible, pre‐retrieval donor blood samples must be used when a LXM is required (Grade 1B).

Objective 2. Overcome concerns around quality assurance and accuracy of deceased donor HLA typing and virtual crossmatching strategies. It is now recognized that alloantibodies can be stimulated by all the classical, polymorphic HLA proteins (HLA‐A, ‐B, ‐C, ‐DRB1/3/4/5, ‐DQA1, DQB1, DPA1 and –DPB1) (Duquesnoy, Marrari et al., 2014a, 2014b). Donor typing to this degree is therefore necessary to enable universal assessment of HLA antibody compatibility and adoption of the VXM, even though, in many cases, this may not be necessary because for individual recipients, where the antibody repertoire tends to be limited, the minimum required HLA typing for safe transplantation would correspond to the same loci as those against which the antibodies are reacting.

In 2017, there was 0.8% error rate in the HLA types used for deceased donor allocation in the United Kingdom (ODT, KAG ODT, KA2018) and in 2/17 cases the errors affected the allocation sequence. In 2018, the error rate was 0.3% and none of the errors affected the allocation sequence (ODT KAG, 2019).

### 3.2 Recommendations

1. We recommend that full HLA typing (HLA‐A, ‐B, ‐C, ‐DRB1/3/4/5, ‐DQA1, DQB1, DPA1 and –DPB1) is generally required for crossmatch interpretation (Tait et al., 2013). This is more extensive than the current minimum level of HLA resolution required for UK deceased donor allocation (ODT datasheet DAT2885/3, 2019) (Grade 1A). However, it is self‐evident that all‐inclusive, unambiguous and the highest resolution HLA typing widens the safe use of the VXM as well as improving the reliable interpretation of crossmatches in general.
2. In order to correctly classify a negative VXM or identify a false‐positive LXM result, typing for HLA loci corresponding to those represented in the recipient's antibody profile must be available (Alzahrani et al., 2019; Tambur et al., 2010) (Grade 1A).
3. Where a recipient has uncharacterized HLA‐specific antibodies, or has a defined antibody but there is an incomplete donor HLA type (in relation to a recipient's antibody specificities, e.g. recipient has allele‐specific antibodies), or where the intended recipient has known donor HLA‐specific antibodies, a pre‐transplant LXM must be performed (Grade 1B).
4. A retrospective LXM (e.g. using cells from the donor spleen or lymph node) may be undertaken when a pre‐transplant VXM has been used. However, if an audit of sufficient cases shows concordance between the initial VXMs and subsequent LXMs then the latter can be safely omitted (Grade 2B).

Objective 3. Address issues relating to donor sample availability and quantity. The timeliness of reporting a prospective crossmatch will depend on early taking of donor blood samples arranged in advance between the laboratory, clinical and donation teams. There are many demands for donor blood samples and anecdotal evidence suggests that excessive blood taking may be harmful to the donor and compromise their donated organs.

### 3.3 Recommendations

1. We recommend that peripheral blood for crossmatch testing must only be requested and sent on demand. Therefore, when a VXM is to be used, crossmatch samples must not be taken from the donor pre‐transplant (Grade 1B).
2. When PBLs for LXM are required, up to a maximum of 40 ml of peripheral blood in EDTA should be requested unless there are exceptional circumstances. In this scenario, the H&I laboratory can request a volume as required for locally validated procedures (Grade 1B).
3. Pre‐retrieval crossmatch donor blood must be taken and sent as expeditiously as possible (Grade 1B).
4. Spleen or lymph node samples must be taken at the time of organ retrieval in all cases (e.g. to verify a VXM by performing a retrospective XM, or to enable LXM if the organ is transferred to secondary accepting unit and VXM cannot be performed) (Grade 1A).

It should be noted that geographical considerations may need to be taken into account when considering feasibility of use of PBLs for LXM, namely time and cost taken to ship the samples, particularly where long distances are involved.

Objective 4. Ensure fairness and equity for all potential transplant recipients as immunologically high‐risk cases tend to wait the longest for a transplant. The interpretation of a crossmatch in such cases tends to be complex, requiring more information. A comprehensive and up‐to‐date HLA serological history, according to national and other guidelines (BSHI & BTS Guideline, 2015; Tait et al., 2013) is therefore a prerequisite for an equitable approach where using a crossmatch to allow a transplant to proceed.

### 3.4 Recommendations

1. We recommend that no patient must be advantaged or disadvantaged because of tractability of the use of a particular type of XM or crossmatch policy (Grade 1A).
2. The crossmatch test should be regarded as an enabler of safe transplantation, rather than primarily a tool to prevent transplantation, particularly for sensitized patients and local policy developed with this in mind (Bingaman et al., 2008) (Grade 2B).

Objective 5. When undertaking virtual crossmatching, there must be a full understanding of the potential risks involved to ensure patient safety is not compromised. All involved must understand that the profiles and levels of HLA‐specific antibodies in individual patients can change and the VXM is currently limited to testing compatibility for HLA. The LXM can detect non‐HLA antibodies, which may or may not be clinically relevant; at the moment, there is insufficient evidence to guide either way. Other donor‐derived target cells can be used to detect non‐HLA, donor‐specific antibodies (e.g. endothelial cell precursors; Zitzner et al., 2013).

### 3.5 Recommendations

1. We recommend that if laboratories are not using HLA antibody results from a sample collected on the day of evaluation, they must have a rigorous mechanism for identifying any potential sensitizing events or changes in immunosuppressive therapy since the date of the last tested sample (Grade 1A). This can be achieved through the development of good working relationships between the clinical team and the laboratory, and the process documented in local policy.
2. Non‐HLA donor‐specific antibodies are outside the scope of this guidance (Grade 2D).

## AUTHOR CONTRIBUTIONS

All authors participated in the working group, contributed to these guidelines, and have read and approved the final manuscript. SP and DB prepared the following text.

## References

[iji12558-bib-0001] Alzahrani, M. , Qahtani, Z. , Harbi, H. , Kebasi, S. , Essa, O. , & Al Attas, R. (2019). Virtual crossmatch: Reality of perception. Transplantation Proceedings, 51(2), 488–491. 30879574 10.1016/j.transproceed.2019.01.005

[iji12558-bib-0002] Atkins, D. , Best, D. , Briss, P. A. , Eccles, M. , Falck‐Ytter, Y. , Flottorp, S. , Guyatt, G. H. , Harbour, R. T. , Haugh, M. C. , Henry, D. , Hill, S. , Jaeschke, R. , Leng, G. , Liberati, A. , Magrini, N. , Mason, J. , Middleton, P. , Mrukowicz, J. , O'Connell, D. , … Zaza, S. (2004). GRADE Working Group 2004 Grading quality of evidence and strength of recommendations. BMJ (Clinical Research Ed.), 328(7454), 1490. 10.1136/bmj.328.7454.1490 PMC428525 15205295

[iji12558-bib-0003] Bingaman, A. W. , Murphey, C. L. , Palma‐Vargas, J. , & Wright, F. (2008). A virtual crossmatch protocol significantly increases access of highly sensitized patients to deceased donor kidney transplantation. Transplantation, 86(12), 1864–1868. 10.1097/TP.0b013e318191404c 19104435

[iji12558-bib-0004] Breimer, M. E. , Rydberg, L. , Jackson, A. M. , Lucas, D. P. , Zachary, A. A. , Melancon, J. K. , Von Visger, J. , Pelletier, R. , Saidman, S. L. , Williams, W. W. , Holgersson, J. , Tydén, G. , Klintmalm, G. K. , Coultrup, S. , Sumitran‐Holgersson, S. , & Grufman, P. (2009). Multicenter evaluation of a novel endothelial cell crossmatch test in kidney transplantation. Transplantation, 87(4), 549–556. 10.1097/TP.0b013e3181949d4e 19307793

[iji12558-bib-0005] BSHI & BTS Guideline . The detection and characterisation of clinically relevant antibodies in allotransplantation. July (2015). https://bts.org.uk/wp‐content/uploads/2016/09/06_BTS_BSHI_Antibodies‐1.pdf

[iji12558-bib-0006] Callaghan, C. J. , Mumford, L. , Pankhurst, L. , Baker, R. J. , Bradley, J. A. , & Watson, C. J. E. (2017). Early outcomes of the new UK deceased donor kidney fast‐track offering scheme. Transplantation, 101(12), 2888–2897. 10.1097/TP.0000000000001860 28640070

[iji12558-bib-0007] Collaborative Transplant Study (CTS) K14101‐0820 www.ctstransplant.org

[iji12558-bib-0008] Collaborative Transplant Study (CTS) K14102‐0820 www.ctstransplant.org

[iji12558-bib-0009] Courtney, A. E. , & Maxwell, A. P. (2009). The challenge of doing what is right in renal transplantation: Balancing equity and utility. Nephron Clinical Practice, 111(1), c62–c68. 10.1159/000180121 19060499

[iji12558-bib-0010] Daniel, V. , Sadeghi, M. , Suesal, C. , Scherer, S. , Tran, H. , Gombos, P. , Trojan, K. , Morath, C. , & Opelz, G. (2016). Clinical relevance of preformed IgG and IgM antibodies against donor endothelial progenitor cells in recipients of living donor kidney grafts. Clinical Transplantation, 30(2), 124–130. 10.1111/ctr.12665 26537026

[iji12558-bib-0011] De Santis, D. , Truong, L. , Martinez, P. , & D'Orsogna, L. (2020). Rapid high‐resolution HLA genotyping by MinION Oxford nanopore sequencing for deceased donor organ allocation. HLA, 96(2), 141–162. 10.1111/tan.13901 32274854

[iji12558-bib-0012] Duquesnoy, R. J. , Marrari, M. , Mulder, A. , da Mata Sousa, L. C. D. , Da Silva, A. S. , & Do Monte, S. J. H. (2014a). First report on the antibody verification of HLA‐ABC epitopes recorded in the website‐based HLA Epitope Registry. Tissue Antigens, 83(6), 391–400. 10.1111/tan.12341 24828056

[iji12558-bib-0013] Duquesnoy, R. J. , Marrari, M. , Tambur, A. R. , Mulder, A. , da Mata Sousa, L. C. D. , da Silva, A. S. , & do Monte, S. J. H. (2014b). First report on the antibody verification of HLA‐DR, HLA‐DQ and HLA‐DP epitopes recorded in the HLA Epitope Registry. Human Immunology, 75(11), 1097–1103. 10.1016/j.humimm.2014.09.012 25305456

[iji12558-bib-0014] Jani, V. , Ingulli, E. , Mekeel, K. , & Morris, G. P. (2017). Root cause analysis of limitations of virtual crossmatch for kidney allocation to highly‐sensitized patients. Human Immunology, 78(2), 72–79. 10.1016/j.humimm.2016.11.003 27864154

[iji12558-bib-0015] Jaramillo, A. , Reddy, K. S. , & Heilman, R. L. (2020). Using the virtual crossmatch to allow for safer and more efficient kidney transplantation of highly sensitized patients. Transplantation, 104(6), 1121–1122. 10.1097/TP.0000000000002925 31449189

[iji12558-bib-0016] Kang, H. , Yoo, J. , Lee, S. Y. , & Oh, E. J. (2021). Causes of positive pretransplant crossmatches in the absence of donor‐specific anti‐human leukocyte antigen antibodies: A single‐center experience. Annals of Laboratory Medicine, 41(4), 429–435. 10.3343/alm.2021.41.4.429 33536364 PMC7884190

[iji12558-bib-0017] Kardol‐Hoefnagel, T. , & Otten, H. G. (2021). A comprehensive overview of the clinical relevance and treatment options for antibody‐mediated rejection associated with non‐HLA antibodies. Transplantation, 105(7), 1459. 10.1097/TP.0000000000003551 33208690 PMC8221725

[iji12558-bib-0018] Key, T. , Carter, V. , Day, S. , Goodwin, P. , Goodwin, J. , Harmer, A. , Knight, A. , Mather, F. , McKane, W. , Poles, A. , & Rigg, K. (2019). Human neutrophil antibodies associated with early and chronic antibody mediated rejection in kidney transplant recipients. Journal of Nephrology & Renal Transplantation Science, 2(2), 81–84.

[iji12558-bib-0019] Kolonko, A. , Ziaja, J. , Król, R. , Chudek, J. , Sekta, S. , Siekiera, U. , Cierpka, L. , & Wie̢cek, A. (2011). Impact of early lymph node procurement to facilitate histocompatibility testing on long‐term cadaveric kidney graft survival. Transplantation Proceedings, 43(8), 2875–2878. 21996177 10.1016/j.transproceed.2011.07.015

[iji12558-bib-0020] Kumar, S. , Doss, S. A. , Stephen, S. , Pratheeba, M. , Jeyaseelan, L. , & Daniel, D. (2021). The challenge of using the virtual crossmatch as a singular tool for the detection of Anti‐HLA antibodies—A study from a tertiary care institute from South India. Transplant Immunology, 65, 101349. 10.1016/j.trim.2020.101349 33127497

[iji12558-bib-0021] Meier‐Kriesche, H.‐U. , & Kaplan, B. (2002). Waiting time on dialysis as the strongest modifiable risk factor for renal transplant outcomes: A paired donor kidney analysis. Transplantation, 74(10), 1377–1381. 10.1097/00007890-200211270-00005 12451234

[iji12558-bib-0022] Middleton, D. , Jones, J. , & Lowe, D. (2014). Nothing's perfect: The art of defining HLA‐specific antibodies. Transplant Immunology, 30(4), 115–121. 10.1016/j.trim.2014.02.003 24582684

[iji12558-bib-0023] Morris, A. B. , Sullivan, H. C. , Krummey, S. M. , Gebel, H. M. , & Bray, R. A. (2019). Out with the old, in with the new: Virtual versus physical crossmatching in the modern era. HLA, 94(6), 471–481. ODT Statistics and reports. https://www.odt.nhs.uk/statistics‐and‐reports/ 10.1111/tan.13693 31515937 PMC7341023

[iji12558-bib-0024] ODT Datasheet DAT2885/3 (2019). https://nhsbtdbe.blob.core.windows.net/umbraco‐assets‐corp/16945/dat2885‐minimum‐resolution‐for‐reporting‐donor‐recipient‐hla‐types‐table‐1.pdf

[iji12558-bib-0025] ODT KAG (2018). https://nhsbtdbe.blob.core.windows.net/umbraco‐assets‐corp/15706/june‐2018‐kag‐minutes‐approved‐271118.pdf

[iji12558-bib-0026] ODT KAG (2019). https://nhsbtdbe.blob.core.windows.net/umbraco‐assets‐corp/16408/kag_minutes_0619.pdf

[iji12558-bib-0027] ODT Policy POL186/10 (2019). https://nhsbtdbe.blob.core.windows.net/umbraco‐assets‐corp/16915/kidney‐allocation‐policy‐pol186.pdf

[iji12558-bib-0028] Patel, R. , & Terasaki, P. I. (1969). Significance of the positive crossmatch test in kidney transplantation. New England Journal of Medicine, 280(14), 735–739. 10.1056/NEJM196904032801401 4886455

[iji12558-bib-0029] Perico, N. , Cattaneo, D. , Sayegh, M. H. , & Remuzzi, G. (2004). Delayed graft function in kidney transplantation. The Lancet, 364(9447), 1814–1827. 10.1016/S0140-6736(04)17406-0 15541456

[iji12558-bib-0030] Rohan, V. S. , Pilch, N. , Moussa, O. , Nadig, S. N. , Dubay, D. , Baliga, P. K. , & Taber, D. J. (2020). Virtual crossmatching in kidney transplantation: The wait is over. Journal of the American College of Surgeons, 230(4), 373–379. 10.1016/j.jamcollsurg.2019.12.031 32035182

[iji12558-bib-0031] Roll, G. R. , Webber, A. B. , Gae, D. H. , Laszik, Z. , Tavakol, M. , Mayen, L. , Cunniffe, K. , Syed, S. , Hirose, R. , Freise, C. , Feng, S. , Roberts, J. P. , Ascher, N. L. , Stock, P. G. , & Rajalingam, R. (2020). A virtual crossmatch‐based strategy facilitates sharing of deceased donor kidneys for highly sensitized recipients. Transplantation, 104(6), 1239–1245. 10.1097/TP.0000000000002924 31449187

[iji12558-bib-0032] Salahudeen, A. K. , Haider, N. , & May, W. (2004). Cold ischemia and the reduced long‐term survival of cadaveric renal allografts. Kidney International, 65(2), 713–718. 10.1111/j.1523-1755.2004.00416.x 14717946

[iji12558-bib-0033] Shaw, J. , Gibson, I. W. , Wiebe, C. , Houston, D. S. , Koulack, J. , Rush, D. , Nickerson, P. , & Ho, J. (2019). Hyperacute antibody‐mediated rejection associated with red blood cell antibodies. Transplantation Direct, 5(8), e477. 10.1097/TXD.0000000000000925 31576373 PMC6708630

[iji12558-bib-0034] Shrestha, S. , Bradbury, L. , Boal, M. , Blackmur, J. P. , Watson, C. J. E. , Taylor, C. J. , Forsythe, J. L. R. , Johnson, R. , & Marson, L. P. (2016). Logistical factors influencing cold ischemia times in deceased donor kidney transplants. Transplantation, 100(2), 422–428. 10.1097/TP.0000000000000844 26262505

[iji12558-bib-0035] Siddiqi, N. , McBride, M. A. , & Hariharan, S. (2004). Similar risk profiles for post‐transplant renal dysfunction and long‐term graft failure: UNOS/OPTN database analysis. Kidney International, 65(5), 1906–1913. 10.1111/j.1523-1755.2004.00589.x 15086934

[iji12558-bib-0036] South, A. M. , & Grimm, P. C. (2016). Transplant immuno‐diagnostics: Crossmatch and antigen detection. Pediatric Nephrology, 31(6), 897–905. 10.1007/s00467-015-3145-z 26139577 PMC5650740

[iji12558-bib-0037] Stockton, J. D. , Nieto, T. , Wroe, E. , Poles, A. , Inston, N. , Briggs, D. , & Beggs, A. D. (2020). Rapid, highly accurate and cost‐effective open‐source simultaneous complete HLA typing and phasing of class I and II alleles using nanopore sequencing. HLA, 96(2), 163–178. 10.1111/tan.13926 32419382

[iji12558-bib-0038] Su, X. , Zenios, S. A. , Chakkera, H. , Milford, E. L. , & Chertow, G. M. (2004). Diminishing significance of HLA matching in kidney transplantation. American Journal of Transplantation, 4(9), 1501–1508. 10.1111/j.1600-6143.2004.00535.x 15307838

[iji12558-bib-0039] Sullivan, H. C. , Dean, C. L. , Liwski, R. S. , Biswas, S. , Goodman, A. L. , Krummey, S. , Gebel, H. M. , & Bray, R. A. (2018). (F) Utility of the physical crossmatch for living donor evaluations in the age of the virtual crossmatch. Human Immunology, 79(10), 711–715. 10.1016/j.humimm.2018.08.001 30081064

[iji12558-bib-0040] Summers, D. M. , Johnson, R. J. , Allen, J. , Fuggle, S. V. , Collett, D. , Watson, C. J. , & Bradley, J. A. (2010). Analysis of factors that affect outcome after transplantation of kidneys donated after cardiac death in the UK: A cohort study. The Lancet, 376(9749), 1303–1311. 10.1016/S0140-6736(10)60827-6 20727576

[iji12558-bib-0041] Tait, B. D. , Süsal, C. , Gebel, H. M. , Nickerson, P. W. , Zachary, A. A. , Claas, F. H. J. , Reed, E. F. , Bray, R. A. , Campbell, P. , Chapman, J. R. , Coates, P. T. , Colvin, R. B. , Cozzi, E. , Doxiadis, I. I. N. , Fuggle, S. V. , Gill, J. , Glotz, D. , Lachmann, N. , Mohanakumar, T. , … Opelz, G. (2013). Consensus guidelines on the testing and clinical management issues associated with HLA and non‐HLA antibodies in transplantation. Transplantation, 95(1), 19–47. 10.1097/TP.0b013e31827a19cc 23238534

[iji12558-bib-0042] Tambur, A. R. , Leventhal, J. R. , Friedewald, J. J. , & Ramon, D. S. (2010). The complexity of human leukocyte antigen (HLA)‐DQ antibodies and its effect on virtual crossmatching. Transplantation, 90(10), 1117–1124. 10.1097/TP.0b013e3181f89c6d 20847715

[iji12558-bib-0043] Taylor, C. J. , Smith, S. I. , Morgan, C. H. , Stephenson, S. , Key, T. , Jones, P. , Watson, C. , Jacques, B. , Welsh, K. I. , & Bradley, J. A. (2000). Selective omission of the donor cross‐match before renal transplantation: Efficacy, safety and effects on cold storage time. Transplantation, 69(5), 719–723. 10.1097/00007890-200003150-00008 10755516

[iji12558-bib-0044] Taylor, C. J. , Kosmoliaptsis, V. , Sharples, L. D. , Prezzi, D. , Morgan, C. H. , Key, T. , Chaudhry, A. N. , Amin, I. , Clatworthy, M. R. , Butler, A. J. , Watson, C. J. E. , & Bradley, J. A. (2010). Ten‐year experience of selective omission of the pretransplant crossmatch test in deceased donor kidney transplantation. Transplantation, 89(2), 185–193. 10.1097/TP.0b013e3181c926f2 20098281

[iji12558-bib-0045] Turner, D. , Battle, R. , Akbarzad‐Yousefi, A. , & Little, A.‐M. (2019). The omission of the “wet” pre‐transplant crossmatch in renal transplant centres in Scotland. HLA, 94(1), 3–10. 10.1111/tan.13558 31025501

[iji12558-bib-0046] Uhlig, K. , Macleod, A. , Craig, J. , Lau, J. , Levey, A. S. , Levin, A. , Moist, L. , Steinberg, E. , Walker, R. , Wanner, C. , Lameire, N. , & Eknoyan, G. (2006). Grading evidence and recommendations for clinical practice guidelines in nephrology. A position statement from Kidney Disease: Improving Global Outcomes (KDIGO). Kidney International, 70(12), 2058–2065. 10.1038/sj.ki.5001875 17003817

[iji12558-bib-0047] Vacher‐Coponat, H. , Purgus, R. , Indreies, M. , Moal, V. , Luciani, H. , Lechevallier, E. , Delaporte, V. , Luccioni, A. , Julian, H. , Reviron, D. , Dussol, B. , & Berland, Y. (2007). Cold ischemia time in renal transplantation is reduced by a timesheet in a French transplant center. Transplantation, 83(5), 561–565. 10.1097/01.tp.0000253757.14344.7f 17353774

[iji12558-bib-0048] Valentin, M. O. , Ruiz, J. C. , Vega, R. , Martín, C. , Matesanz, R. , Lozano, J. J. G. , & Dalmau, A. G. (2016). Implementation of a national priority allocation system for hypersensitized patients in Spain, based on virtual crossmatch: Initial results. Transplantation Proceedings, 48(9), 2871–2875. 27932095 10.1016/j.transproceed.2016.09.024

[iji12558-bib-0049] Watson, C. J. E. , Johnson, R. J. , & Mumford, L. (2020). Overview of the evolution of the UK kidney allocation schemes. Current Transplantation Reports, 7(2), 140–144. 10.1007/s40472-020-00270-6

[iji12558-bib-0050] Wisse, B. W. , Kamburova, E. G. , Joosten, I. , Allebes, W. A. , van der Meer, A. , Hilbrands, L. B. , Baas, M. C. , Spierings, E. , Hack, C. E. , van Reekum, F. E. , & van Zuilen, A. D. (2019). Toward a sensible single‐antigen bead cutoff based on kidney graft survival. Transplantation, 103(4), 789. 10.1097/TP.0000000000002357 30106794 PMC6430591

[iji12558-bib-0051] Zecher, D. , Bach, C. , Preiss, A. , Staudner, C. , Utpatel, K. , Evert, M. , Jung, B. , Bergler, T. , Böger, C. A. , Spriewald, B. M. , & Banas, B. (2018). Analysis of luminex‐based algorithms to define unacceptable HLA antibodies in CDC‐crossmatch negative kidney transplant recipients. Transplantation, 102(6), 969–977. 10.1097/TP.0000000000002129 29470350

[iji12558-bib-0052] Zitzner, J. R. , Shah, S. , Jie, C. , Wegner, W. , Tambur, A. R. , & Friedewald, J. J. (2013). A prospective study evaluating the role of donor‐specific anti‐endothelial crossmatch (XM‐ONE assay) in predicting living donor kidney transplant outcome. Human Immunology, 74(11), 1431–1436. 10.1016/j.humimm.2013.06.007 23777928

